# Impact and Perspectives of Pediatric Dental Care during the COVID-19 Pandemic Regarding Unvaccinated Children: A Cross-Sectional Survey

**DOI:** 10.3390/ijerph182212117

**Published:** 2021-11-18

**Authors:** Nelly Schulz-Weidner, Maximiliane Amelie Schlenz, Norbert Krämer, Sarra Boukhobza, Katrin Bekes

**Affiliations:** 1 Dental Clinic, Department of Pediatric Dentistry, Justus Liebig University, Schlangenzahl 14, 35392 Giessen, Germany; nobert.kraemer@dentist.med.uni-giessen.de; 2 Dental Clinic, Department of Prosthodontics, Justus Liebig University, Schlangenzahl 14, 35392 Giessen, Germany; maximiliane.a.schlenz@dentist.med.uni-giessen.de; 3 Department of Pediatric Dentistry, University Clinic of Dentistry, Medical University of Vienna, 1090 Vienna, Austria; sarra.boukhobza@meduniwien.ac.at (S.B.); katrin.bekes@meduniwien.ac.at (K.B.)

**Keywords:** dentistry, questionnaire, COVID-19, coronavirus, pandemics, patient care, pediatric dentistry, dentists

## Abstract

The pandemic caused by the COVID-19 virus has led to enormous challenges in pediatric dental care. In contrast to adults, many children are without any symptoms of disease if infected with COVID-19 and are not vaccinated. The aim of this study was to conduct an inquiry into the perspective of pediatric dentists in Germany towards the impact of COVID-19 on daily patient care during the lockdowns caused by the pandemic. All members of the Germany Society of Pediatric Dentistry were invited to participate in an online survey. The questionnaire focused on five topics: safety measures, well-being/knowledge, patient care, prospects, and demographic data. A total of 549 pediatric dentists participated (58.11% females, 8.2% males, 0.18% inter/diverse, and 33.51% no answer). In total, 75.2% of the dental clinics were open during the first lockdown (LD1) and 78.1% during the second lockdown (LD2). In LD1, only 22.2% were operating at full capacity, while 40.1% were open with restrictions, and 11.8% only offered emergency treatment. In LD2, 71.2% of practices were operating again and resumed offering all treatment methods. A significant decrease in pediatric patients was reported due to the fact of COVID-19. Furthermore, measures, such as oral hygiene and recall appointments, were reduced. Measures that were performed after the lockdown were mainly aerosol-generating procedures and extractions as well as general anesthesia or sedation. The increased willingness to treat in the second lockdown has shown that pediatric dentists have adapted to the pandemic conditions, which seems to be of great importance, especially in view of the problem of unvaccinated children.

## 1. Introduction

The COVID-19 pandemic has put health care services across the globe under enormous stress [1,2,3]. In particular, pediatric dentists were faced with unique and varying challenges during the different waves of the pandemic [4]. Scarce reliable information at the beginning led to sweeping lockdowns and social distancing which, in turn, meant that many patients did not show up for dental check-ups with their children, leading to a significant increase in caries lesions [5,6,7]. As of January 2021, with several vaccines being available to limit the spread of COVID-19, some of the measures have eased. However, mutations and so called “anti-vaxxers”, a significant part of the population who show an unwillingness to become vaccinated, will affect pediatric dentists and other health professionals for some time to come [8].

During the first lockdown, there was a significant amount of uncertainty around COVID-19, from its etiology, to transmission pathways, to the likelihood of fatal outcomes or long-term side effects and treatment methods. At the beginning of the pandemic, it was unclear whether the risk of infection was even more pronounced for pediatric dentists. General practitioners faced a risk from patients who were not in the incubation period or who tried to conceal or were not aware of their infection [9,10]. Initially, it was unclear whether and how children were vulnerable to COVID-19, as children who contract the virus show no symptoms or are prone to have a mild course of the disease [11]. Therefore, most of these infections remained undetected, and the question of the infectivity of these asymptomatic children was yet be answered. Studies have since shown that the few children who do show symptoms have a very similar course regarding the disease as adults [12,13]. Furthermore, researchers found that child-to-child transmissions of COVID-19, for example, in schools, is unlikely and not the main cause for the infection in children [14]. In Germany, the rate of infected children, especially during the first lockdowns, was very low and constituted only an insignificant part of the total number of infections [15]. Recent data have reinforced the conception that COVID-19 in children is not as severe regarding both the symptomatic progression as well as long-term side effects and fatality rate as compared to adults; however, especially young children and infants remain vulnerable to this disease and may act as transmitters [16,17,18].

Against the background of these uncertainties and since there were no binding legal requirements in Germany at that time, this article, among other things, explores the question of how pediatric dentists in Germany reacted to the first wave of the pandemic, which subsided at the end of May 2020. Of particular interest were the concrete personal solutions to questions, such as the procurement of the necessary personal protective equipment and other protective measures, in situations of uncertainty. Knowing how pediatric dentists have dealt with and are still dealing with these challenges will enable decision-makers to adapt their communication and policy recommendations. Pediatric care presents particular challenges for pediatric dentists due to the factors mentioned above including asymptomatic children with COVID-19 infection and a lack of vaccination opportunities; therefore, this study evaluated concerns about self-infection and infection of others and also queried the impact of COVID-19 measures on workflows and procedures in private practices. As the base of evidence on the modes of transmission, infectiousness of children, and long-term side effects has grown and becomes more reliable, this study also aimed to investigate whether this led to an adjustment of service provision in private practices during the second lockdown.

## 2. Materials and Methods

### 2.1. Online Survey

All pediatric dentists (*n* = 1830) who were members of the German Society of Pediatric Dentistry (GSPD(DGKiZ)) and practice in Germany were invited to participate in the online survey.

The questionnaire was designed in cooperation with the Teaching Evaluation Service Centre of the JLU and provided using an established online survey tool (LimeSurvey, Hamburg, Germany, Supplementary Materials). The generated link was shared by the office of the DGKiZ via email.

The survey included multiple-choice questions asking about COVID-19 and pediatric dentistry including additional safety measures (14 questions); well-being, safety, and anxiety about self-infection or infecting others (8 questions); patient care process (4 questions); knowledge about COVID-19 (7 questions); prospects from the pandemic (3 questions); demographic data (8 questions). For systematic evaluation, a five-point Likert scale [19] was used, and abstention was allowed. The survey was evaluated anonymously, and the study was conducted in accordance with the ethical standards of the Institutional Review Board and the local ethics committee of the JLU (AZ 29/21).

The data collected were completely anonymous, and it is not possible to trace the identity of the dentists. The survey was conducted on 14 December 2020. After 7 and 28 days, a reminder was sent to all potential participants. The survey was closed on 22 January 2021. Only completed questionnaires were included.

### 2.2. Statistical Analysis

Statistical analyses were performed using SPSS Statistics (version 26; IBM, Armonk, NY, USA). The distribution of responses is presented as the mean and standard deviation. Furthermore, data on anxiety were analyzed. In detail, one-way ANOVA was performed for anxiety analysis of self-infection and infection of other persons. Descriptive statistics were used to summarize and analyze the data. Absolute and relative frequencies and measures of central tendency and dispersion were calculated according to the scale level and distribution of each variable. Spearman’s correlations were used between impairments of additional infection control measures.

## 3. Results

### 3.1. Sociodemographic Data and Profession-Related Characteristics

Out of the 1830 members of the DGKiZ, who were invited to participate in the survey, 549 dentists responded (30% response rate). Of these, 319 were female, 45 male, 1 inter/diverse, and 184 did not respond. The mean age was 44.58 ± 9.38 years (range 25–74 years). Years of dental practice ranged from 1 to 50 years with a mean of 17.9 ± 9.16 years.

In terms of belonging to a risk group, this was reported by 13.3% (*n* = 73) of colleagues: 5.8% (*n* = 32) due to the fact of chronic diseases, 7.1% due to the fact of age (*n* = 38), 1.46% due to the fact of obesity (*n* = 8), 0.7% due to the fact of smoking (*n* = 4), 0.56% due to the fact of immunosuppression (*n* = 3), 0.6% due to the fact of pregnancy (*n* = 3), autoimmune deficiency (one response), and atopic dermatitis and asthma (one response).

Other reasons for risk provided were patient contact, the profession itself, especially aerosol- generating procedures, and the treatment of children itself due to the fact of their lack of symptoms.

### 3.2. Patient Care

During the 1st lockdown (LD1) from March 2020 to May 2020, 75.2% of the dental clinics were open, whereas 78.1% of the dental clinics were open during the 2nd lockdown (LD2) from December 2020 to April 2021. In LD1, only 22.2% were fully operational, while 40.1% were open on a restricted basis and 11.8% opened for emergency treatment only. This changed in LD2, where 71.2% of practices were again operating all measures, whereas only 6.7% operated on a restricted basis. Regarding these restricted measures, it can be seen that oral hygiene appointments and dental cleanings in restricted operation increased significantly again in the second lockdown.

**Figure 1:**
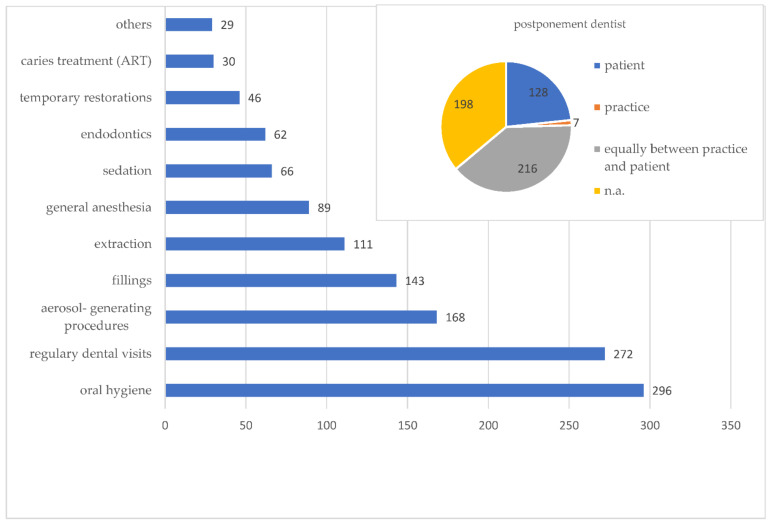
Number (*n*) and type of postponed measures with reasons for cancelled appointments.

In terms of appointment rescheduling, 63.9% (*n* = 51) of colleagues reported that treatment appointments were postponed due to the fact of COVID-19, and 23.3% of the appointments were cancelled by patients (only 1.28% from the practice side). The most common reason was the patient’s concern about being infected with COVID-19 in the dental clinic. Most of these postponed measures were prophylaxis appointments (53.9%), followed by dental check-ups (49.5%) and caries therapy with rotary instruments (30.6%) as well as filling therapies (26%) (Figure 1).

### 3.3. Sources of Knowledge Gain/Information Regarding Patient Care during Pandemic

**Figure 2:**
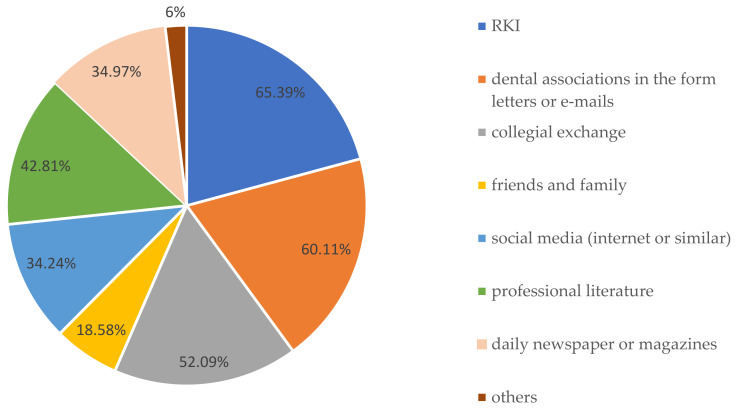
Information sources regarding COVID-19.

Only 55.01% (*n* = 302) of colleagues stated that they felt well prepared to manage patient care during the COVID-19 pandemic. They were mainly informed using Robert Koch Institute (RKI) information (65.39%), information from several dental associations (60.11%), and professional literature (42.8%), followed by collegial exchange (52%). Social media, including podcasts, also played a role for over 34% of the participants. In total, 15% indicated interest in training on COVID-19 infection control (Figure 2).

### 3.4. Infection Control 

**Figure 3:**
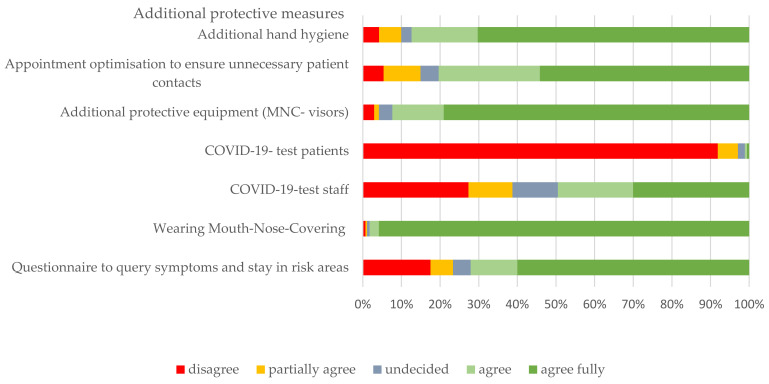
Participants’ assessment of the usefulness of the additional protective measures applied.

Regarding additional infection control measures, the majority found personal protective equipment to be useful. Questionnaires on symptom and stay in risk areas as well as appointment optimization to avoid unnecessary patient contact and additional hand hygiene were also found to be good. A COVID-19 test before every treatment was indicated as less useful; this was also considered useful more than half the time for the staff (Figure 3).

### 3.5. Change in Practice Routine Compared to before the Pandemic

**Figure 4:**
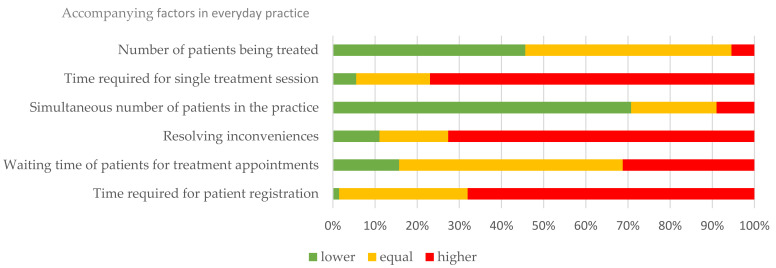
Accompanying changes in daily routine during COVID-19 compared to before the pandemic.

For accompanying factors in everyday practice regarding the negative circumstances surrounding the pandemic, 45.9% of colleagues stated that more time had to be spent on patient registration. However, in the majority of cases, there was no increase in waiting time for appointments and for individual treatment sessions but mainly inconvenience for patients (such as increased waiting time in the registration area). The number of patients treated in the practice decreased by 48.8% and by 31.9% for altered treatment (Figure 4).

Regarding the presence of parents during treatment, 52% of colleagues stated that they only allowed one parent into the practice with them. For aerosol-generating procedures, 62.3% reported wearing FFP-2, followed by visors. Most of the colleagues obtained personal protective equipment from the dental depot (62.85%), followed by pharmacies (10.6%), collegial contacts (9%), and dental associations (9.8%).

Considering possible impairments in additional infection control measures, most dentists agreed that they were affected by limited communication and lack of facial expressions due to the additional protective equipment (e.g., visors and masks). Thereby, the impairment due to the measures of the additional protective equipment was significant in terms of limited communication with the patients and missing facial expressions (Table 1).

### 3.6. COVID-19 Infection

**Figure 5:**
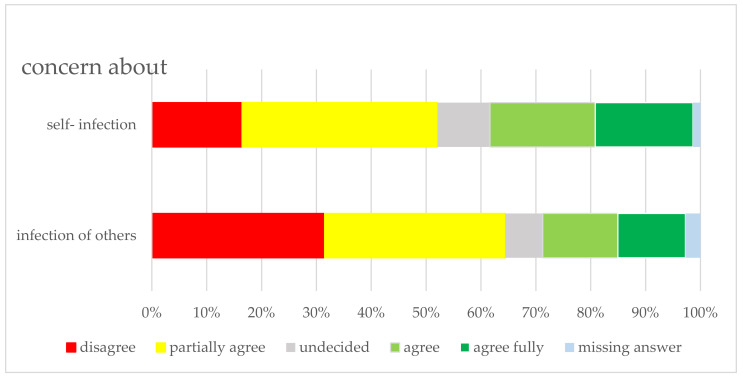
Participants’ assessment of the concern of self-infection and infection of others.

Regarding possible self-infection, 43.5% (*n* = 239) of the respondents stated that they had worried at least once about contracting COVID-19; only 2.3% (*n* = 13) became ill. Seven (1.28%) were infected in their private environment at home and three in private practice (professional environment); one was infected via a co-worker and one while shopping (one no answer). Figure 5 shows respondents’ statements about self-infection and concern about infecting others. Respondents who assigned themselves to a risk group (13.3%) had a significantly higher fear of self-infection (*p* < 0.05).

**Figure 6:**
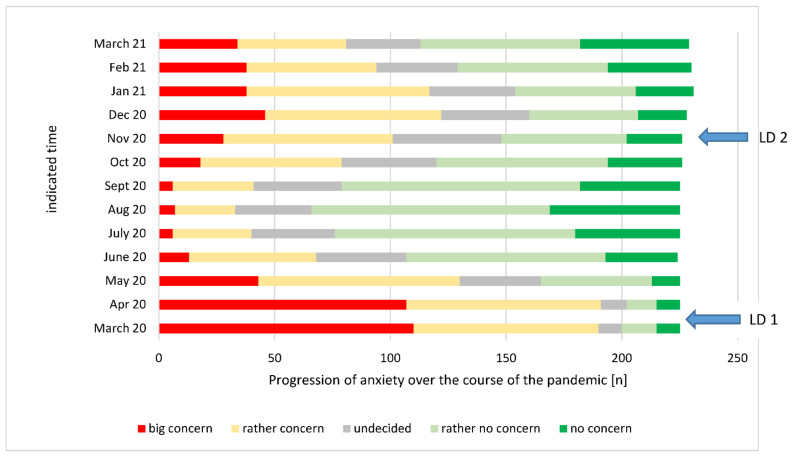
Reported anxiety for self-infection on a Likert scale during the period from March 2020 to March 2021.

Using our questionnaire, we also retrospectively analyzed the period from March 2020 to March 2021 on the anxiety scale (measured according to a Likert scale). An impressive increase in anxiety was clearly demonstrated during the first and second lockdowns (Figure 6).

In detail, the majority did not feel uncomfortable treating during the COVID-19 pandemic and stated their desire to support regular patient treatment with increased hygiene and protective measures and not only emergency treatment. Taking into account additional time-consuming protective measures that complicate daily patient care and communication, 60.29% of respondents thought it was good to not only provide emergency care. Regardless of these abovementioned aspects, the majority said that they would maintain additional protective measures after the COVID-19 pandemic with respect to other airborne infectious diseases (e.g., tuberculosis) depending on the situation.

## 4. Discussion

The study shows that the range of treatments offered was adjusted by pediatric dentists during the course of the pandemic. While only 22.2% were fully functional in the first LD1, this changed in LD2, where 71.2% of the practices were again performing all measures. With regard to postponed measures, it was found that oral hygiene appointments and dental cleanings and regular dental check-ups were most commonly postponed, followed by aerosol-generating procedures. These results are also worrying because an increase in the incidence of deciduous tooth decay during the COVID-19 pandemic has already been reported [20]. This shows the importance of continuous pediatric dental care and the need for preventive dental care during the COVID-19 pandemic, which is in agreement with a study showing more self-reported caries during the lockdown in Wuhan [21], representing the importance of prophylaxis and treatment even in epidemics.

In contrast to adult treatment, where a return to normality in everyday treatment can be expected due to the possibility of vaccination, this does not appear to be returning in pediatric dental practice, as no change in everyday practice can be expected due to the lack of vaccination possibilities. The difficulty of testing young children due to the lack of cooperation and the possible asymptomatic COVID-19 infected children show that pediatric dentists in particular have to cope with the challenge of an increased risk of self-infection. Considering that 43% of colleagues, at least once, reported having been concerned about self-infection with COVID-19 in the practice, this seems to be a serious problem and underlines the importance of sufficient infection control. Kathree et al. [22] showed that measures focusing, on the one hand, on infrastructure and organization and, on the other hand, on personal protective equipment (PPE) for staff and patients to reduce infection were implemented more or less strongly. This confirms our results which also showed different levels of acceptance and implementation of PPE. This fact may also be related to the uncertainty about how to adapt the new infrastructure and organization, which has already been described showing the difficulty of implementation of safety measures in everyday practice [23].

These additional protective measures, such as visors and FFP-2 masks, which appear to be important in the protection against infection and are considered as crucial in infection control, were stated to be a burden for 37% of the pediatric dentists, especially due to the limited communication aspect with patients as well as a lack of facial expressions, which are an important factor in the behavioral management for successful treatment of children. Nevertheless, the majority of respondents stated they did not feel uncomfortable while treating during the COVID-19 pandemic and would support regular patient treatment with increased hygiene and protective measures beyond emergency treatment only.

In our study, only 55% of respondents felt well prepared to manage patient care during the COVID-19 pandemic, which does not appear to be advantageous, especially in view of increasing early childhood caries (ECCs). These findings are in accordance with the literature. Dentists need to actively and regularly seek out and use reputable and reliable sources of information for dealing with pediatric patients that are appropriate for their own region and circumstances [24,25]. Nevertheless, our results suggest that dentists have adapted their new daily practice to the pandemic situation. In LD2, 71.2% of dentists operated fully, and it seems that they were able to adapt their daily practice to the pandemic despite the stated challenges, which is particularly encouraging considering that there is still no end to the pandemic in sight.

As all participants were informed that the data collection was completely anonymous and did not allow for tracing, response bias was prevented. The questions were scored using a Likert scale, which is the standard procedure for surveys in the medical field [19]. A limitation of this study is that data collection was completed within one month. Considering possible rapid changes in regulations and infection rates, this short period of data collection could influence the results. In particular, the retrospective question on fear of contracting COVID-19 might not be as accurate. Therefore, the results should be interpreted with an awareness of the retrospective nature.

As expected, the response rate resulted in a smaller sample size. However, we could not target the non-responders because the survey was anonymous. In addition, we may not have reached dentists who work in pediatric dentistry but are not members of the DGKiZ; we believe that pediatric dentists identify with the DGKiZ and that this group should only be a small proportion. The majority (58.1%) of participants were female. However, as pediatric dentists in Germany are predominantly female and in view of the fact that survey respondents were from all federal states, it can be assumed that our results are representative for pediatric dentists in Germany.

Overall, the study shows that pediatric dentists have adapted well to the new patient environment in the course of the pandemic, which appears to be of great importance for further pediatric dental patient care, especially with regard to unvaccinated children.

## 5. Conclusions

A strong decrease in pediatric patients was reported due to the fact of COVID-19. Moreover, measurements, such as oral hygiene and recall appointments, were reduced. Throughout the course of the pandemic, however, it became apparent that pediatric dentists returned to normal patient care under increased safety measures and showed that they were able to maintain patient care despite the additional workload and adverse effects.

## Figures and Tables

**Table 1:** Spearman’s correlations between impairments of additional infection control measures.

		Impairment Limited Communication	Impairment Missing Facial Expression
Impairment missing facial expression	Correlation coefficient	0.534	
	*p* (two-tailed)	<0.001	
	*n*	402	
Impairment due to the additional infection control measures	Correlation coefficient	−0.005	0.030
	*p* (two-tailed)	0.913	0.543
	*n*	404	405

## Data Availability

The data sets used in this article are available from the corresponding author upon reasonable request.

## Supplementary Materials

The following are available online at https://www.mdpi.com/article/10.3390/ijerph182212117/s1, Questionnaire S1.
