# Supplementary material for: Impact and Perspectives of Pediatric Dental Care during the COVID-19 Pandemic Regarding Unvaccinated Children: A Cross-Sectional Survey
Source: Int J Environ Res Public Health. 2021 Nov 18;18(22):12117. doi: 10.3390/ijerph182212117 (PMC8618805; doi:10.3390/ijerph182212117)
Supplement: Supplementary file 1 [file ijerph-18-12117-s001.zip › ijerph-1430290-supplementary.pdf]

S1 Questionnaire

A COVID-19 and pediatric dentistry

A1. Were you open for business during the first lockdown?

- Yes
- No

A2. How had you opened your practice?

- Fully
- restricted
- only for emergency treatment

A3. Which measures did you offer?

- Oral hygiene measures and prophylaxis
- Check ups
- Endodontics in the primary dentition
- Caries treatment with rotary instruments
- Caries treatment using the ART technique
- temporary fillings
- definitive fillings
- extraction
- others

A4. Were you open for business during the second lockdown?

- Yes
- No

A5. How had you opened your practice?

- Fully
- restricted
- only for emergency treatment

A6. Which measures did you offer?

- Oral hygiene measures and prophylaxis
- only controls
- Endodontics in the primary dentition
- Caries treatment with rotary instruments
- Caries treatment using the ART technique
- temporary fillings
- definitive fillings
- extraction
- others

A7. Were appointments cancelled due to COVID-19?

- Yes
- No

A8. Who postponed the appointment?

- Patient
- Practice
- Equally patient and practice

A9. Why was the appointment postponed?

- Patient was concerned about possible infection with COVID-19 in the practice.
- Family/friends advised against visiting the practice due to increased risk of infection.
- doctor advised against visit because patient belongs to risk group.
- Other

A10. What type of appointment did this involve?

- Check up
- Oral hygiene, prophylaxis
- Endodontics in the primary dentition
- Caries treatment with rotary instruments
- Caries treatment using the ART technique
- temporary fillings
- definitive fillings
- extraction
- treatment under general anaesthesia
- nitrous oxid sedation
- others

A11. As a pediatric dentist, do you consider yourself well prepared to manage patient care in COVID-19 times?

- yes
- no

A12. What are your sources of information about the COVID-19 infection?

- RKI
- Dental associations in the form of letters or e-mails
- Collegial exchange
- Friends and family members
- Social media (internet or similar)
- Specialist literature
- Daily newspaper or magazine
- Others

A13. Are you aware of guidelines/guidelines on COVID-19 infection?

- yes
- no

A14. Have you attended a training course on COVID-19 infection control?

- yes
- no
- I would be interested in a training course
- I would not be interested in training

A15.

	strongly disagree	disagree	neutral	agree	strongly agree	no answer
I would like to see training on dental treatment in times of a pandemic.	○	○	○	○	○	○

B. Well-being/safety

B1. To what extent do the following statements apply to you about current patient care during the COVID-19 pandemic?

Please select the appropriate answer for each point:

	strongly disagree	disagree	neutral	agree	strongly agree	No answer
I think it is good that regular patient treatment is maintained with increased hygiene and protective measures and not only emergency treatment is provided	○	○	○	○	○	○
I am afraid to infect MYSELF.	○	○	○	○	○	○

	strongly disagree	disagree	neutral	agree	strongly agree	No answer
I am afraid to infect OTHERS.	○	○	○	○	○	○
I feel uncomfortable.	○	○	○	○	○	○
In my opinion, the patients feel uncomfortable.	○	○	○	○	○	○

B2. Which of the following measures do you take with regard to Infection control in the practice?

Please select the appropriate answer for each point:

	strongly disagree	disagree	neutral	agree	strongly agree	No answer
questionnaire to query symptoms and stay in risk areas	○	○	○	○	○	○
wearing mouth-to-nose coverings throughout the whole practice	○	○	○	○	○	○
Corona test staff	○	○	○	○	○	○
Corona test before each treatment	○	○	○	○	○	○
additional protective equipment (visors,	○	○	○	○	○	○

	strongly disagree	disagree	neutral	agree	strongly agree	No answer
FFP2 masks etc.) on the treatment side						
Appointment optimisation to ensure unnecessary patient contacts	○	○	○	○	○	○
additional hand hygiene	○	○	○	○	○	○
others	○	○	○	○	○	○

B3. Which of the following aspects affect you?

Please select the appropriate answer for each point:

	strongly disagree	disagree	neutral	agree	strongly agree	no answer
limited communication due to additional protective equipment (e.g. visor, mask)	○	○	○	○	○	○
longer waiting time due to additional measures	○	○	○	○	○	○
lack of facial expression due to additional protective equipment (e.g. visor, mask)	○	○	○	○	○	○
others	○	○	○	○	○	○

B4. Were you at least at one time afraid of being infected with COVID-19 at the practice?

- o Yes
- o No

B5. Please indicate for each month how much fear you had of contracting COVID-19 at the practice.

	Large fear	rather fear	undecided	rather no fear	none fear
march	○	○	○	○	○
apr	○	○	○	○	○
may	○	○	○	○	○
jun	○	○	○	○	○
jul	○	○	○	○	○
aug	○	○	○	○	○
sep	○	○	○	○	○
oct	○	○	○	○	○
nov	○	○	○	○	○
dec	○	○	○	○	○
jan	○	○	○	○	○
feb	○	○	○	○	○

B6. Do you think, that all hygiene rules related to infection control were followed in the COVID-19 pandemic in the following areas?

	never	sometimes	often	More often	always
patient registration	○	○	○	○	○
waiting rooms	○	○	○	○	○
treatment /Dentists´ room	○	○	○	○	○

B7. Do you perceive the increased hygiene rules and additional protective measures (e.g., wearing mouth/nose covering in the building, hand hygiene, keeping your distance, regular ventilation) as a burden during your stay at the practice?

- Yes, the measures are a burden for me.
- No, the additional measures are not a problem for me.

B8. Arrange the following actions in descending order of how they impact you the most (highest impact at the top, lowest impact at the bottom)

wear mouth-nose covering permanently indoors.	○
ventilate regularly	○
keeping distance from other people	○
limited use of elevator	○
hand hygiene	○
handlers' faces covered by additional protective measures (e.g. visors, masks)	○

C. Procedure

C1. Please assess whether and to what extent pediatric patient care in the practice SINCE the COVID-19 pandemic with regard to the following aspects has changed compared to before the pandemic:

	shorter	unchanged	longer	I cannot judge
time required for patient organisation	○	○	○	○
Waiting time for treatment in general	○	○	○	○
Occurrence of inconveniences (e.g. waiting outside, "traffic" in the registration area)	○	○	○	○
Number of patients in the practice at the same time	○	○	○	○
time required for individual treatment sessions	○	○	○	○
Numbers of patients being treated	○	○	○	○

C2. If you have to work with aerosol producing measures, which Mouth Covering do you use?

- FFP3
- FFP2
- Medical Mouth-nose covering
- Protective shield/visor
- Medical Mouth-nose covering and protective shield/visor

C3. How did you get protective equipment (masks etc.)?

- Dental depot
- Pharmacy

- Collegial contacts
- others

C4. How do you currently organise your daily practice with regard to parental support in patient care?

- Child may only come with one parent
- only 1 patient/parent in the waiting room
- several patients/parents in the waiting room, but with sufficient distance between them
- Patient appointments at long intervals without contact with other households

D. Knowledge

D1. The virus which has caused the current pandemic is called

- COVID-19
- new SARS-2 mutation
- severe acute respiratory syndrome (SARS)
- 2019-nCOV
- Severe acute respiratory syndrome (SARS-CoV-2)

D2. Which age groups are affected by COVID-19?

(Multiple answers possible)

- under 5 years
- between 5 and 14 years
- between 15 and 24 years
- between 25 and 34 years
- between 35 and 44 years
- between 45 and 54 years
- between 55 and 64 years
- between 65 and 79 years
- 80-100 years old

D3. COVID-19 can be transmitted by

- a handshake
- Coughing and sneezing
- Touching infected surfaces such as table and pet knobs
- others

D4. In my opinion, the most common symptom described with a COVID-19-infection is...

- Fever
- Cough
- Sore throat
- Diarrhoea
- Difficulty breathing
- Loss of smell and taste

D5. In my opinion, the incubation period of COVID-19 is ...

- 1-14 days
- 1-7 days
- 2-5 days
- 1-14 hours

D6. In my opinion, isolation is called....

- the separation of those who are ill and suspected of being infected with COVID 19 infected
- the separation of those who are not yet ill but have been exposed to COVID 19
- the same as quarantine

D7. What is the main feature of a rapid antibody test kit?

- Confirms that the person tested has been exposed to COVID-19 infection.
- Confirms that the person tested has been exposed to COVID-19 infection.
- Confirms that the person tested is not infected with COVID-19
- Test result takes more than 48 hours
- Test shows positive result within 24 hours of infection

<u>Part E: Outlook</u>

E1. For how long (month/s) do you think the COVID-19 pandemic will continue to change our patient care in the dental clinic?

E2. Would you be in favor of maintaining the additional protective measures after the COVID-19 pandemic with regard to other airborne infectious diseases (e.g. tuberculosis)?

Yes, always.	○
Yes, depending on the situation (e.g. in the winter months or when there is an increased risk).	○
I cannot judge	○
no	○

E3. What additional hygiene and protective measures would you recommend? (Multiple selection possible)

questionnaire to query symptoms and stay in risk areas.	○
one-way street system and entry system	○
wearing face coverings throughout the whole attendance	○
Corona test before each treatment	○
additional protective equipment (visors, FFP2 masks, etc.) on the practioner´s side	○
structural partitioning of the treatment boxes into individual "treatment rooms"	○
additional hand hygiene	○
others	○

<u>Part F Final questions</u>

F1. What is your gender?

Please select only one of the following answers:

- male
- female
- inter / diverse

F2.Please state your age in years.

F3. How long have you been practising dentistry?

F4. Do you belong to the risk group with regard to a possible COVID-19 infection?

- ○ Yes
- ○ No

F5. Why do you belong to the risk group? (Multiple selection possible)

Age +50 years	○
chronic disease (e.g. diabetes, high blood pressure, heart, lung, liver or kidney disease)	○
smoker	○
immunosuppression	○
obesity	○
pregnancy	○
others	○

F6. Have you ever been diagnosed with COVID-19?

- ○ Yes
- ○ No

F7: Where do you think you became infected with COVID-19?

in private environment (at home)	○
in private environment (vacation)	○
in professional environment (practice)	○
in professional environment (professional trip)	○
in professional environment (medical appointment)	○

in professional environment (dental appointment)	○
in professional environment (stay in hospital)	○
others	○

F8. What else would you like to tell us? Use the space for further suggestions and comments
